# Protobacco Media Exposure and Youth Susceptibility to Smoking Cigarettes, Cigarette Experimentation, and Current Tobacco Use among US Youth

**DOI:** 10.1371/journal.pone.0134734

**Published:** 2015-08-26

**Authors:** Erika B. Fulmer, Torsten B. Neilands, Shanta R. Dube, Nicole M. Kuiper, Rene A. Arrazola, Stanton A. Glantz

**Affiliations:** 1 Office on Smoking and Health, Centers for Disease Control and Prevention, 4770 Buford Highway, N.E., MS F-79, Atlanta, Georgia, 30341, United States of America; 2 Center for AIDS Prevention Studies (CAPS), Department of Medicine, University of California San Francisco, 50 Beale Street, Suite 1300, San Francisco, California, 94105, United States of America; 3 Epidemiology and Biostatistics, School of Public Health, Georgia State University, 140 Decatur Street, Suite 848, Atlanta, Georgia, 30303, United States of America; 4 Center for Tobacco Control Research and Education, Department of Medicine, University of California San Francisco, 530 Parnassus Avenue, San Francisco, California, 94143–1390, United States of America; Brown University, UNITED STATES

## Abstract

**Purpose:**

Youth are exposed to many types of protobacco influences, including smoking in movies, which has been shown to cause initiation. This study investigates associations between different channels of protobacco media and susceptibility to smoking cigarettes, cigarette experimentation, and current tobacco use among US middle and high school students.

**Methods:**

By using data from the 2012 National Youth Tobacco Survey, structural equation modeling was performed in 2013. The analyses examined exposure to tobacco use in different channels of protobacco media on smoking susceptibility, experimentation, and current tobacco use, accounting for perceived peer tobacco use.

**Results:**

In 2012, 27.9% of respondents were never-smokers who reported being susceptible to trying cigarette smoking. Cigarette experimentation increased from 6.3% in 6th grade to 37.1% in 12th grade. Likewise, current tobacco use increased from 5.2% in 6th grade to 33.2% in 12th grade. Structural equation modeling supported a model in which current tobacco use is associated with exposure to static advertising through perception of peer use, and by exposure to tobacco use depicted on TV and in movies, both directly and through perception of peer use. Exposure to static advertising appears to directly increase smoking susceptibility but indirectly (through increased perceptions of peer use) to increase cigarette experimentation. Models that explicitly incorporate peer use as a mediator can better discern the direct and indirect effects of exposure to static advertising on youth tobacco use initiation.

**Conclusions:**

These findings underscore the importance of reducing youth exposure to smoking in TV, movies, and static advertising.

## Introduction

Smoking tobacco causes diseases in most organs of the body [1]. The severity of smoking-related diseases increases with duration and level of exposure to cigarette use, which makes starting smoking during adolescence a problem. [2,3]. Given current prevalence, 5.6 million children younger than 18 years will die prematurely due to smoking [1]. In addition to cigarette smoking, use of other tobacco products is increasing among youth [3,4].

Tobacco industry actions to promote tobacco use such as tobacco advertising, promotions, and product marketing at the point of sale, as well as exposure to smoking onscreen in motion pictures, cause tobacco use among youth in complex and dynamic ways [3]. These protobacco influences work at multiple points across social and environmental contexts to affect youth tobacco related beliefs, intentions and behaviors. [5] In addition to the direct effects of protobacco influences on adolescent tobacco use, related increases in peer and parental smoking linked to tobacco promotional activities and exposure to on-screen smoking act as mediating variables between advertising and youth tobacco use [3,6–8].

Changing social norms around tobacco use is a focus of tobacco control efforts. However, youth in the US are still exposed to protobacco influences, including smoking depicted in movies and TV. Exposure to smoking in movies causes youth to start smoking—the more onscreen exposure to smoking, the more likely youth are to smoke [3,9]. Research demonstrates that exposure to smoking in movies is a stronger predictor of future smoking than other types of tobacco marketing and is associated with greater susceptibility, the perception that most adults smoke, and reduced time to first use [10–12]. Studies also suggest that the effect of exposure to smoking in movies is mediated through peer affiliations, in that youth who view more of these movies show an increase in affiliation with peers who smoke [6,7]. Although less studied, smoking on TV is another significant source of exposure [13].

The 1998 Master Settlement Agreement (MSA) resolved litigation among 46 states against the 5 largest US cigarette companies [14]. In 2006, US District Judge Gladys Kessler ruled these companies were racketeers that created an illegal enterprise to defraud the public [15,16]. As part of the MSA, participating tobacco companies agreed not to pay for *branded* tobacco product placements in movies, TV, and other entertainment venues; whether the MSA restricted *unbranded* tobacco placement is unresolved. Additionally, the MSA has no effect on nonparticipating companies or operations outside the US. Despite MSA marketing restrictions, US tobacco companies spent $9.2 billion, or $25 million per day, in 2012 to promote domestic tobacco products [17,18].

Studies consistently show that a high percentage of youth are exposed to, aware of, and recall tobacco advertising, including static tobacco advertising in newspapers, magazines, retail stores, and the Internet [19–21]. This exposure increases susceptibility and causes onset and continuation of smoking among youth [3,19,21–23]. However, the association between smoking and other channels of protobacco media is uncertain. To address this research gap, the current study uses a large, nationally representative survey of both US middle and high school students to examine associations among exposures to tobacco use in TV and movies; static tobacco advertising and susceptibility; and experimentation and current tobacco use, accounting for indirect mediating effects, including perceived peer tobacco use.

## Methods

### Data Source

The National Youth Tobacco Survey (NYTS) is a voluntary, anonymous, self-administered survey of US middle and high school students in grades 6–12 [24]. Conducted approximately every 2 years, the survey measures susceptibility to trying cigarettes, experimentation with tobacco products, current tobacco use, tobacco-related knowledge and attitudes, and recall exposure to protobacco media and advertising, as well as a range of demographic variables [25]. The NYTS uses a three-stage cluster sample design to produce a nationally representative sample. Probabilistic sampling methods are employed without replacement at all stages including selection of Primary Sampling Units (PSUs) (a county, or a group of small counties, or part of a large county) within each stratum, of schools within each PSU, and of students within each school. The sample is stratified by racial/ethnic composition and urban versus non-urban status at the primary stage. Additionally, the sampling frame includes all public, parochial, and other private and charter school students enrolled in grades 6 to 12 in the 50 US states and the District of Columbia [24]. This paper uses data collected in 2012 (school response rate = 80.3%, student response rate = 91.7%, overall response rate = 73.6%; this yielded an n = 24,658 students) and analyzed in 2013. With an overall response rate of greater than 60%, the data was able to be weighted to reflect the initial probabilities of selection and non-response patterns, to mitigate large variations in sampling weights, and to post-stratify the data to known sampling frame characteristics.

### Ethics Statement

Protocols were approved by the Centers for Disease Control and Prevention Institutional Review Board-G (IRB00000188, CDC Protocol #4118). The approved IRB protocols included written parental permission and oral consent for all respondents to ensure student anonymity. Student participation was voluntary and anonymous and subsequent data were analyzed anonymously.

### Measures

#### Susceptibility

Susceptibility to initiate cigarette use was assessed by first asking respondents whether they had ever smoked a cigarette, even one or two puffs. Never-smokers who were susceptible to smoking were defined as those who responded in any way other than “no” to the question, “Do you think you will smoke a cigarette in the next year?” and responded in any way other than “definitely not” to either question: “Do you think you will smoke a cigarette soon?” or “If one of your best friends would offer you a cigarette, would you smoke it?” [26].

#### Experimentation

Cigarette experimentation was defined as having puffed on a cigarette at least once but not having smoked a total of 100 lifetime cigarettes.

#### Current Tobacco Use

Current tobacco use was defined as using on at least 1 day in the past 30 days any of the following tobacco products: cigarettes, cigars, smokeless tobacco, pipe, bidis, kreteks, snus, hookah, roll-your-own cigarettes, dissolvable tobacco products, electronic cigarettes, or some other new tobacco product. Current tobacco use includes both experimenters and established smokers; a respondent who reported smoking a cigarette on one or more days in the past 30 days but not having smoked a total of 100 lifetime cigarettes would be categorized as both an experimenter and a current user, but not an established smoker.

#### Protobacco Media Exposure

Exposure to tobacco use in TV and movies was assessed by asking respondents, “When you watch TV or go to the movies, how often do you see actors and actresses using cigarettes or other tobacco products?” Exposure to static ads was measured by using the following three questions: (1) “When you go to a convenience store, supermarket, or gas station, how often do you see any ads or promotions for cigarettes or other tobacco products?” (2) “When you read newspapers or magazines, how often do you see any ads or promotions for cigarettes or other tobacco products?” and (3) “When you are using the Internet, how often do you see any ads or promotions for cigarettes or other tobacco products?” Respondents answering ‘always,’ ‘most of the time,’ ‘sometimes,’ ‘rarely,’ and ‘never’ to any of the items were included in the analyses. Although Internet content can be dynamic and interactive, the decision to include Internet ads as a static advertising channel was made based on the current wording of the NYTS item and historically static advertising and promotional content located on corporate websites. As tobacco industry Internet advertising becomes increasingly dynamic, more study will be needed to clarify the effects of exposure to this medium.

#### Peer Tobacco Use Perceptions

Perception of peer tobacco use was measured by using two questions: (1) “Out of every 10 students in your grade at school, how many do you think smoke cigarettes?” and (2) “Out of every 10 students in your grade at school, how many do you think use tobacco products other than cigarettes?” Response options ranged from 0 to 10.

#### Respondent Characteristics

Respondents reported their sex (1 = female; 2 = male), race/ethnicity (1 = non-Hispanic white; 2 = non-Hispanic black; 3 = Hispanic; 4 = non-Hispanic other), the number of different products used by family members and those living with respondent (scale ranging from 0 to 4), and the respondent’s grade in school (grades 6–12).

### Statistical Analysis

Data were adjusted for nonresponses and weighted to produce national estimates. All analyses accounted for the complex survey design [24]. The data set contained 24,658 cases; 4 cases were missing all variables considered in the models and excluded, resulting in a final sample of n = 24,654, including n = 17,888 never-smokers. One-way frequency tables were generated for the following outcome variables: (1) never-smokers susceptible to smoking; (2) cigarette experimenters; and (3) current tobacco users, both overall and by grade, sex, and race/ethnicity, by using SAS-Callable SUDAAN 11.0.

**Fig 1:**
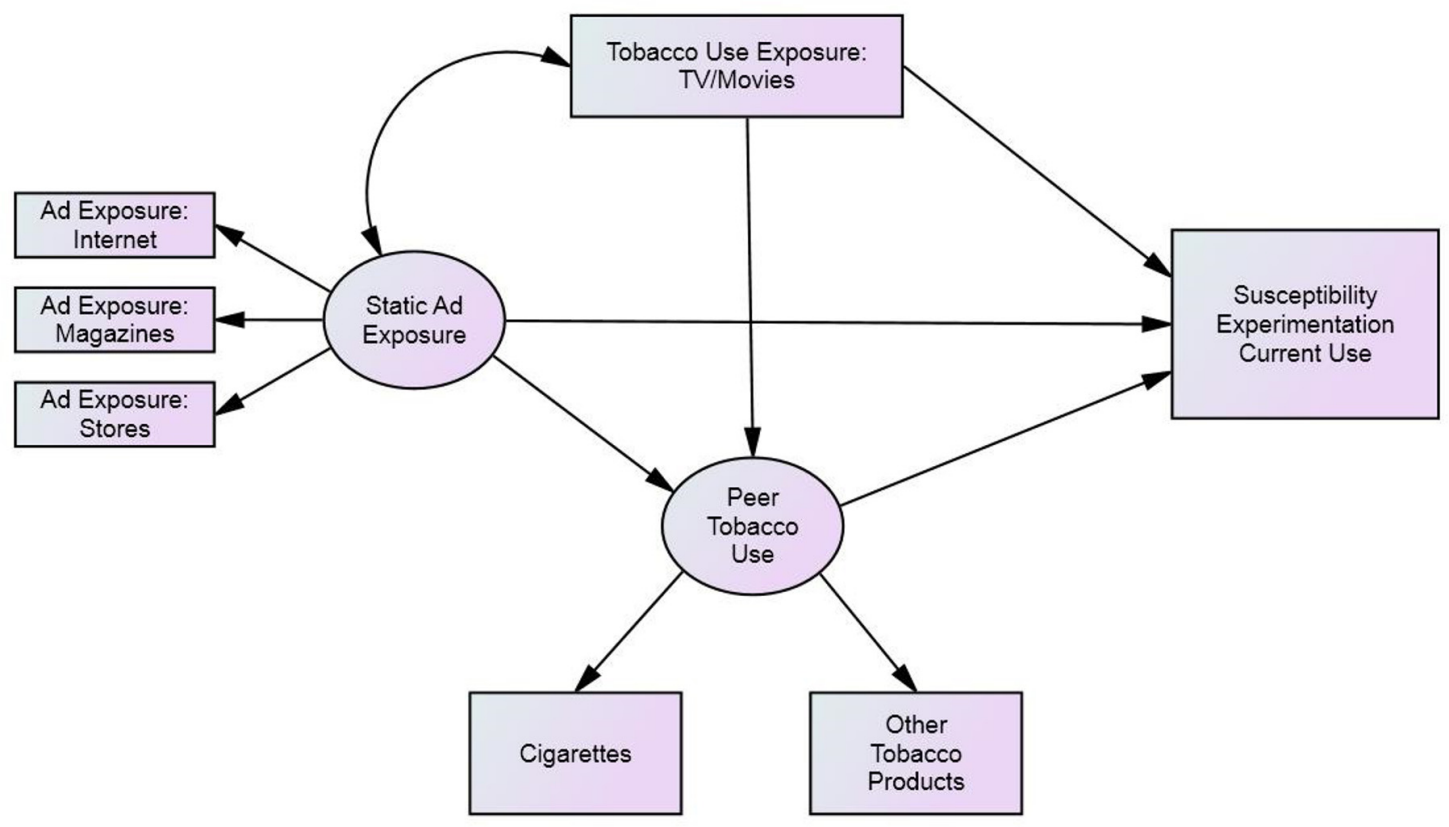
Direct and indirect effects of protobacco media exposure on susceptibility, experimentation, and current use. Conceptual model demonstrating the direct and indirect effects of exposure to static ads and tobacco use in TV and movies on susceptibility to smoking cigarettes, cigarette experimentation, and current tobacco use among US youth. Variables presented in rectangular boxes are observed, whereas unmeasured (latent) variables, including static ad exposure and peer tobacco use, are represented within ellipses. Straight lines with a unidirectional arrow depict direct relationships between variables. Curved lines with bidirectional arrows represent covariation between variables. Covariates that were included in the SEM analyses and tables, but not depicted in the diagram: household member tobacco use, sex, grade in school, black race/ethnicity, Hispanic race/ethnicity, and other ethnicity.

To better understand patterns of correlation among variables, structural equation models (SEM) (Fig 1) were fitted to the data, separately for each of the three primary outcomes by using M*plus* version 7. Structural equation models are extensions of regression models that may incorporate observed and unobserved variables and multiple equations [27]. Structural equation models are represented by path diagrams in which unobserved latent variables are represented by circles or ovals and observed variables are represented by squares or rectangles. Arrows connect the variables in the path diagram with single-headed arrows representing a regression relationship with the direction of the arrow signifying the direction of the effect. Dual-headed arrows represent bi-directional correlation [28]. For instance, in Fig 1 peer tobacco use is a latent variable that is regressed onto static ad exposure, which is also a latent variable, whereas static ad exposure is correlated with the observed tobacco use exposure variable.

Based on multiple longitudinal studies [6,7,10], we specified the structural equation model such that static ad exposure and dynamic exposure to tobacco use in TV and movies influenced perceptions of peers’ tobacco use, which in turn affected susceptibility, experimentation and current tobacco use. Separate structural equation models were estimated for each of these three outcomes. Each SEM included a latent static ad recall exposure variable measured by recall of newspaper or magazine ad exposure (among those who read newspapers or magazines), recall of retail store ad exposure (among those who go to convenience stores or supermarkets or gas stations), and recall of Internet ad exposure (among those who use the Internet). To clarify the role of recall exposure to tobacco use in TV and movies (among those who watch TV or movies) and because TV and movie exposure is qualitatively different than more static advertising, the TV and movie exposure variable was treated as a separate observed explanatory variable. Perception of peer tobacco use was considered in each of the SEM models because preliminary analyses consistently found statistically significant effects suggesting that perception of peer tobacco use mediates the relationship between the explanatory variable and the outcome, subject to the limitations of cross-sectional data. Additional covariates incorporated into the model included: sex, race/ethnicity, grade in school, and number of different tobacco products used by family members and those living with the respondent.

Measurement scales for the latent static tobacco ad exposure and perception of peer tobacco use variables were set on the basis of Internet ad exposure and perception of peers’ use of cigarettes, respectively. Observed endogenous variables with fewer than five categories or strong floor or ceiling effects were treated as ordinal (i.e., newspaper or magazine ad exposure; retail store ad exposure; Internet ad exposure) or binary (i.e., susceptibility to cigarette use; cigarette experimentation; current tobacco use). The variables pertaining to TV and movie tobacco use exposure and perception of peer tobacco use were treated as continuous [29]. The latent variables—static ad exposure and peer tobacco use—were continuous.

Due to the inclusion of binary and ordinal endogenous variables, the SEMs were estimated by using a diagonally weighted least squares (DWLS) estimator based on the multivariate probit distribution [30]. Structural equation modeling relies on a number of statistical tests to determine the adequacy of model fit to the data. In these analyses, global fit was determined by using the χ^2^ test of exact model-data fit with a mean and variance adjustment to the test statistic required when using DWLS estimation to obtain accurate results [29, 30]. Because of the large sample size, trivial model-data fit departures can result in a significant χ^2^ test. Accordingly, approximate fit was assessed by Bentler’s comparative fit index (CFI; [31]), which compares the relative fit of the hypothesized model to a baseline model with no relationships among the variables; the root mean square error of approximation (RMSEA; [32]), which compares the fit of the model in the sample data to what the model’s fit would be in the population if the null hypothesis were true, and the weighted root mean square residual (WRMR; [33]), which represents the average size of the model’s residuals. Simulation studies suggest that if any two of the following three conditions are met, the hypothesized model fits the data well on an approximate basis: CFI ≥ .95, RMSEA ≤ .06, and WRMR ≤ 1.00 [34].

For each direct effect, the unstandardized probit regression coefficient *B* represents the amount of change in the dependent variable per one-unit change in the independent variable. *B*, the 95% CI of *B*, *Z* (the ratio of each parameter estimate to its SE), and the *P*-value (probability level) for *Z* are listed with their corresponding standardized effects, β, representing the standard deviation change in the dependent variable per standard deviation change in the independent variable.

## Results

### Respondent Characteristics by Outcome

In 2012, 27.9% of respondents who never smoked cigarettes reported being susceptible to trying cigarettes during the next year (Table 1). Susceptibility increased by grade starting in 6th grade (24.2%), peaking in 9th grade (33.1%), and decreasing in 12th grade (24.0%), independent of sex. Susceptibility by race/ethnicity was highest among Hispanics (34.1%).

**Table 1:** Demographic characteristics of respondents susceptible to cigarette use, cigarette experimenters, and current tobacco users, NYTS, 2012.

	Never-smokers whoare susceptible tocigarette use ^a^(*n* = 17,888)^b^	Cigaretteexperimentation^c^(*n* = 23,248) ^b^	Current tobacco use^d^(*n* = 23,629) ^b^
	Middle + High School	Middle + High School	Middle + High School
	% (95% CI)	% (95% CI)	% (95% CI)
Total	27.91 (26.99–28.83)	22.15 (20.37–24.02)	17.28 (15.86–18.79)
Grade			
6th	24.18 (21.89–26.63)	6.30 (5.10–7.76)	5.16 (4.24–6.28)
7th	28.39 (26.45–30.41)	11.70 (9.80–13.90)	6.76 (5.41–8.42)
8th	32.00 (29.78–34.29)	18.26 (15.51–21.39)	11.11 (9.28–13.25)
9th	33.12 (30.73–35.60)	24.78 (21.65–28.20)	17.71 (15.28–20.43)
10th	27.27 (24.83–29.86)	28.91 (25.73–32.33)	22.43 (19.64–25.49)
11th	24.70 (22.34–27.22)	33.04 (29.89–36.35)	26.09 (23.62–28.73)
12th	23.95 (21.13–27.02)	37.06 (33.80–40.44)	33.12 (30.38–35.98)
Sex			
Female	26.60 (25.23–28.03)	21.31 (19.31–23.46)	13.42 (12.03–14.95)
Male	29.24 (28.15–30.35)	22.98 (21.16–24.90)	21.02 (19.33–22.81)
Race/Ethnicity			
non-Hispanic white	26.09 (24.77–27.46)	20.45 (18.29–22.79)	17.05 (15.36–18.90)
non-Hispanic black	27.02 (24.80–29.37)	24.21 (20.44–28.43)	17.87 (15.06–21.09)
Hispanic	34.12 (31.94–36.36)	26.58 (23.87–29.47)	18.90 (16.56–21.49)
non-Hispanic Other	26.50 (23.36–29.88)	20.73 (18.27–23.44)	14.33 (11.87–17.20)

NYTS, National Youth Tobacco Survey

^a^ Never-smokers who are susceptible to cigarette use was defined as never tried smoking cigarettes, even one or two puffs and responded other than “definitely not” to the following questions: “Do you think you will smoke a cigarette in the next year?” and “Do you think you will smoke a cigarette soon?” and “If one of your best friends would offer you a cigarette, would you smoke it?”

^b^ Reported *n* based on univariate analyses with missing values excluded.

^c^ Cigarette experimentation was defined as having puffed on a cigarette at least once but not having smoked a total of 100 lifetime cigarettes.

^d^ Current tobacco use was defined as using on at least 1 day in the past 30 days any of the following tobacco products: cigarettes, cigars, smokeless tobacco, pipe, bidis, kreteks, snus, hookah, roll-your-own cigarettes, dissolvable tobacco products, electronic cigarettes, or some other new tobacco product.

Experimentation increased by grade, starting at 6.3% in 6th grade, and increased to 37.1% in 12th grade, with no difference by sex. Experimentation by race/ethnicity was highest among Hispanics (26.6%). Current tobacco use increased by grade, starting at 5.2% in 6th grade, and increased to 33.2% in 12th grade. Current tobacco use was higher among males (21.0%) than females (13.4%), and highest among Hispanics (18.9%).

### Structural Equation Modeling

#### Susceptibility to Cigarette Use Among Never-Smokers

The model χ^2^ test rejected the null hypothesis of exact model-data fit (χ^2^(28) = 213.53, *P* < .0001), but the model fit the data very well on an approximate basis (CFI = .98; RMSEA = .02; WRMR = .99). As shown in Table 2, significant positive direct effects included static advertising exposure and TV and movie exposure on the perception of peer tobacco use. Household member tobacco use, female sex, increasing grade in school, and non-Hispanic black race/ethnicity were also positively associated with perception of peer tobacco use. Susceptibility was positively associated with static ad exposure, family household member tobacco use, male sex, and being Hispanic.

**Table 2:** SEM results: Direct effects, *Susceptibility to Cigarette Use* among middle and high school students, NYTS, 2012^a^.

Outcome Variable	Explanatory Variable	*B* ^b^	95% CI	*Z*	*P*	β ^c^
Internet	Static exposure^d^	1.00	—	—	—	0.77
Newspaper/ magazine	Static exposure	1.02	0.99, 1.05	65.79	< .001	0.78
Retail store	Static exposure	0.77	0.74, 0.80	52.63	< .001	0.60
Cigarettes	Perception of peer tobacco use^e^	1.00	—	—	—	0.87
Noncigarette	Perception of peer tobacco use	1.03	0.98, 1.08	40.34	< .001	0.89
Perception of peer tobacco use	Static exposure	0.68	0.55, 0.82	10.18	< .001	0.21
Perception of peer tobacco use	TV/movie exposure^f^	0.07	0.01, 0.14	2.19	.028	0.03
Perception of peer tobacco use	Household member tobacco use^g^	0.39	0.29, 0.49	7.73	< .001	0.11
Perception of peer tobacco use	Sex	-0.53	-0.63, -0.42	-10.14	< .001	-0.10
Perception of peer tobacco use	Grade	0.44	0.38, 0.50	13.92	< .001	0.34
Perception of peer tobacco use	Black	1.42	0.19, 2.65	2.27	.023	0.19
Perception of peer tobacco use	Hispanic	1.00	-0.32, 2.33	1.48	.138	0.16
Perception of peer tobacco use	Other	-0.37	-4.72, 3.99	-0.17	.869	-0.05
Susceptibility^h^	Static exposure	0.25	0.20, 0.30	9.39	< .001	0.19
Susceptibility	Perception of peer tobacco use	0.01	-0.01, 0.03	1.39	.165	0.03
Susceptibility	TV/movie exposure	0.03	-0.01, 0.06	1.39	.165	0.03
Susceptibility	Household member tobacco use	0.13	0.09, 0.16	7.77	< .001	0.09
Susceptibility	Sex	0.10	0.05, 0.15	3.72	< .001	0.05
Susceptibility	Grade	-0.02	-0.04, 0.001	-1.84	.066	-0.04
Susceptibility	Black	0.05	-0.23, 0.33	0.35	.729	.02
Susceptibility	Hispanic	0.25	0.12, 0.38	3.83	< .001	.10
Susceptibility	Other	-0.01	-0.40, 0.39	-0.04	.970	-.002

SEM, Structural Equation Model

NYTS, National Youth Tobacco Survey

^a^ Multivariate *n* = 17,188 based on all available cases across all variables used in analyses.

^b^ *B* = unstandardized regression coefficient which represents the amount of change in the dependent variable per one-unit change in the independent variable.

^c^ β = standardized regression coefficient, which represents the SD change in the dependent variable per SD change in the independent variable.

^d^ Static exposure was defined as exposure to static tobacco advertisements on the Internet, in newspaper and magazines or retail stores.

^e^ Perception of peer tobacco use measured by student response to the questions (1) “Out of every 10 students in your grade at school, how many do you think smoke cigarettes?” and (2) “Out of every 10 students in your grade at school, how many do you think use tobacco products other than cigarettes?”

^f^ TV and movie exposure was defined as exposure to tobacco use in TV and movies.

^g^ Household member tobacco use was defined as number of tobacco products used by a family member or those living with the respondent.

^h^ Susceptibility was defined as never tried smoking cigarettes, even 1 or 2 puffs and responded in any way other than “no” to the question, “Do you think you will smoke a cigarette in the next year?” and responded in any way other than “definitely not” to either question: “Do you think you will smoke a cigarette soon?” or “If one of your best friends would offer you a cigarette, would you smoke it?”

A significant positive total effect of static ad exposure on susceptibility was observed (B = .26; 95% CI = .21, .31; *P* < .001; β = .20). As shown in Table 2, the direct effect of static ad exposure on susceptibility was positive and significant. However, the indirect effect of static ad exposure on susceptibility through perception of peer tobacco uses was not significant (B = .01; 95% CI = -.003, .02; *P* = .16; β = .01), which suggests that static ad exposure effects on susceptibility did not operate through peer tobacco use.

The total effect of TV and movie tobacco use exposure on susceptibility was not statistically significant (B = .03; 95% CI = -.01, .06; *P* = .15; β = .03). As shown in Table 2, the direct effect of TV and movie tobacco exposure on susceptibility was not significant, nor was the indirect effect of TV and movie exposure on susceptibility through perception of peer tobacco use (B = .001; 95% CI = -.001, .003; *P* = .26; β = .001). Taken collectively, these results indicate that TV and movie tobacco use did not affect susceptibility either directly or indirectly though perception of peer tobacco use.

Changes in susceptibility across grades prompted assessment of the linearity of relationships between grade and each of the three outcome variables. Flexible, nonparametric regression techniques were used to test the linearity of the relationships. There was evidence of weak nonlinearity for the susceptibility outcome (*P* = .06), but not for current tobacco use or cigarette experimentation (cumulative sums of residuals *P* = .42). A sensitivity analysis was performed for the susceptibility SEM model by adding a squared term for grade to the original model. Results mirrored the linear model reported in Table 2 with one exception: the regression of peers on non-Hispanic black race was non-significant (*P* = .51). All other direct, indirect, and total effects yielded identical conclusions.

#### Cigarette Experimentation

The model χ^2^ test rejected the null hypothesis of exact model-data fit (χ^2^ (28) = 402.16, *P* < .0001), but the approximate model statistics indicated satisfactory model-data fit (CFI = .96; RMSEA = .02; WRMR = 1.31). As shown in Table 3, significant positive direct effects included static advertising exposure, household member tobacco use, female sex, and grade. All were positively associated with perception of peer tobacco use. Experimentation was positively associated with household member tobacco use, male sex, and grade.

**Table 3:** SEM results: Direct effects for *Cigarette Experimentation* among middle and high school students, NYTS, 2012^a^.

Outcome Variable	Explanatory Variable	*B* ^b^	95% CI	*Z*	*P*	β ^c^
Internet	Static exposure^d^	1.00	—	—	—	0.76
Newspaper/ magazine	Static exposure	1.06	1.03, 1.08	77.95	< .001	0.80
Retail store	Static exposure	0.75	0.73, 0.78	57.67	< .001	0.57
Cigarettes	Perception of peer tobacco use^e^	1.00	—	—	—	0.87
Noncigarette	Perception of peer tobacco use	1.03	0.97, 1.06	45.39	< .001	0.87
Perception of peer tobacco use	Static exposure	0.79	0.28, 1.29	3.07	.002	0.22
Perception of peer tobacco use	TV/movie exposure^f^	0.07	-0.20, 0.33	0.49	.623	0.03
Perception of peer tobacco use	Household member tobacco use^g^	0.50	0.41, 0.59	10.86	< .001	0.15
Perception of peer tobacco use	Sex	-0.50	-0.60,-.40	-9.89	< .001	-0.09
Perception of peer tobacco use	Grade	0.46	0.31, 0.61	5.99	< .001	0.34
Perception of peer tobacco use	Black	2.07	-9.43, 13.57	0.35	.727	0.26
Perception of peer tobacco use	Hispanic	1.15	-19.54, 21.84	0.11	.913	0.18
Perception of peer tobacco use	Other	-0.52	-38.27, 37.22	-0.03	.978	-0.06
Experimentation^h^	Static exposure	0.10	-0.03, 0.23	1.50	.135	0.08
Experimentation	Perception of peer tobacco use	0.08	-0.01, 0.17	1.68	.096	0.22
Experimentation	TV/movie exposure	0.02	-0.04, 0.08	0.59	.554	0.02
Experimentation	Household member tobacco use	0.26	0.14, 0.38	4.37	< .001	0.21
Experimentation	Sex	0.12	0.06, 0.19	3.78	< .001	0.06
Experimentation	Grade	0.14	0.08, 0.20	4.47	< .001	0.27
Experimentation	Black	-0.12	-3.87, 3.62	-0.06	.950	-0.04
Experimentation	Hispanic	0.67	-3.81, 5.14	0.29	.769	0.27
Experimentation	Other	0.83	-6.75, 8.40	0.21	.830	0.25

SEM, Structural Equation Model

NYTS, National Youth Tobacco Survey

^a^ Multivariate *N* = 24,654 based on all available cases across all variables used in analyses.

^b^ *B* = unstandardized regression coefficient, which represents the amount of change in the dependent variable per one-unit change in the independent variable.

^c^ β = standardized regression coefficient, which represents the SD change in the dependent variable per SD change in the independent variable.

^d^ Static exposure was defined as exposure to static tobacco advertisements on the Internet, in newspaper and magazines or retail stores.

^e^ Perception of peer tobacco use measured by student response to the questions (1) “Out of every 10 students in your grade at school, how many do you think smoke cigarettes?” and (2) “Out of every 10 students in your grade at school, how many do you think use tobacco products other than cigarettes?”

^f^ TV and movie exposure was defined as exposure to tobacco use in TV and movies.

^g^ Household member tobacco use was defined as number of tobacco products used by a family member or those living with the respondent.

^h^ Experimentation was defined as having puffed on a cigarette at least once but not having smoked a total of 100 lifetime cigarettes.

A significant positive total effect of static ad exposure on experimentation was observed (B = .16; 95% CI = .05, .28; *P* = .006; β = .12). As indicated in Table 3, the direct effect of static ad exposure on experimentation was not significant. However, the indirect effect of static ad exposure on experimentation through perception of peer tobacco use was positive and significant (B = .06; 95% CI = .03, .10; *P* = .001; β = .05), which suggests that the effect of ad exposure on experimentation operates exclusively through perception of peer tobacco use.

The total effect of TV and movie tobacco use exposure on experimentation was not statistically significant (B = .02; 95% CI = -.05, .09; *P* = .50; β = .03). The direct effect of TV and movie tobacco use on experimentation in Table 3 was not significant nor was the indirect effect of TV and movie exposure on experimentation through perception of peer tobacco uses (B = .01; 95% CI = -.02, .03; *P* = .70; β = .01). Thus, there was no association observed between TV and movie tobacco use exposure and experimentation.

#### Current Tobacco Use

The model χ^2^ test rejected the null hypothesis of exact model-data fit (χ^2^(28) = 412.68, *P* < .0001), but the approximate model statistics indicated satisfactory model-data fit (CFI = .96; RMSEA = .02; WRMR = 1.32). As shown in Table 4, current tobacco use was positively associated with TV and movie tobacco exposure, perception of peer tobacco use, household member tobacco use, male sex, and grade.

**Table 4:** SEM results: Direct effects for *Current Tobacco Use* among middle and high school students, NYTS, 2012^a^.

Outcome Variable		Explanatory Variable	*B* ^b^	95% CI	*Z*	*P*	β ^c^
Internet	Static exposure^d^		1.00	—	—	—	0.76
Newspaper/ magazine	Static exposure		1.06	1.03, 1.09	80.08	< .001	0.80
Retail store	Static exposure		0.75	0.73, 0.78	57.98	< .001	0.57
Cigarettes	Perception of peer tobacco use^e^		1.00	—	—	—	0.86
Noncigarette	Perception of peer tobacco use		1.05	1.004, 1.09	49.14	< .001	0.89
Perception of peer tobacco use	Static exposure		0.77	0.64, 0.90	11.51	< .001	0.22
Perception of peer tobacco use	TV/movie exposure^f^		0.07	0.01, 0.13	2.44	.015	0.03
Perception of peer tobacco use	Household member tobacco use^g^		0.49	0.38, 0.59	9.09	< .001	0.15
Perception of peer tobacco use	Sex		-0.49	-0.57, -0.41	-11.64	< .001	-0.09
Perception of peer tobacco use	Grade		0.46	0.39, 0.52	14.59	< .001	0.34
Perception of peer tobacco use	Black		1.76	-0.26, 3.79	1.71	.088	0.23
Perception of peer tobacco use	Hispanic		1.19	-0.15, 2.53	1.74	.083	0.19
Perception of peer tobacco use	Other		-0.60	-5.95, 4.75	-0.22	.826	-0.07
Current use^h^	Static exposure		0.03	-0.01, 0.08	1.36	.173	0.02
Current use	Perception of peer tobacco use		.10	0.09, 0.12	12.34	< .001	0.28
Current use	TV/movie exposure		0.04	0.01, 0.06	2.69	.007	0.04
Current use	Household member tobacco use		0.27	0.25, 0.30	20.07	< .001	0.22
Current use	Sex		0.37	0.32, 0.43	14.29	< .001	0.19
Current use	Grade		0.14	0.12, 0.16	14.78	< .001	0.27
Current use	Black		-0.02	-0.28, 0.25	-0.11	.912	-0.01
Current use	Hispanic		0.05	-0.09, 0.20	0.70	.482	0.02
Current use	Other		-0.10	-0.24, 0.05	-1.27	.205	-0.03

SEM, Structural Equation Model

NYTS, National Youth Tobacco Survey

^a^ Multivariate *N* = 24,654 based on all available cases across all variables used in analyses.

^b^ *B* = unstandardized regression coefficient, which represents the amount of change in the dependent variable per one-unit change in the independent variable.

^c^ β = standardized regression coefficient, which represents the SD change in the dependent variable per SD change in the independent variable.

^d^ Static exposure was defined as exposure to static tobacco advertisements on the Internet, in newspaper and magazines or retail stores.

^e^ Perception of peer tobacco use measured by student response to the questions (1) “Out of every 10 students in your grade at school, how many do you think smoke cigarettes?” and (2) “Out of every 10 students in your grade at school, how many do you think use tobacco products other than cigarettes?”

^f^ TV and movie exposure was defined as exposure to tobacco use in TV and movies.

^g^ Household member tobacco use was defined as number of tobacco products used by a family member or those living with the respondent.

^h^ Current use was defined as using on at least 1 day in the past 30 days any of the following tobacco products: cigarettes, cigars, smokeless tobacco, pipe, bidis, kreteks, snus, hookah, roll-your-own cigarettes, dissolvable tobacco products, electronic cigarettes, or some other new tobacco product.

A significant positive total effect of static ad exposure on current tobacco use was observed (B = .11; 95% CI = .07, .15; *P* < .001; β = .08). The direct effect of static ad exposure on current tobacco use listed in Table 4 was not significant. However, the indirect effect of static ad exposure on current tobacco use through perception of peer tobacco use was positive and significant (B = .08; 95% CI = .06, .10; *P* < .001; β = .06). Taken collectively, these results suggest that the effect of static ad exposure on current tobacco use operated exclusively through perception of peer tobacco use.

The total effect of TV and movie tobacco use exposure on current tobacco use was statistically significant (B = .04; 95% CI = .02, .07; *P* = .002; β = .05). As shown in Table 4, the direct effect of TV and movie tobacco use on current tobacco use was significant as was the indirect effect of TV and movie exposure on current tobacco use through perception of peer tobacco use (B = .01; 95% CI = .001, .013; *P* = .02; β = .01). The significance of both the direct effect of TV and movie tobacco use exposure on current tobacco use and the indirect effect of TV and movie tobacco use exposure on current tobacco use via perception of peer tobacco use implies that TV and movie tobacco use exposure’s effect on current tobacco occurs in part through perception of peer tobacco use but also in ways other than via peer tobacco use perceptions.

## Discussion

Youth are exposed to protobacco media influences through numerous channels including static tobacco advertising in newspapers and magazines, in retail stores, and on the Internet, as well as through exposure to tobacco use in TV and movies. Our results provide an important addition to the existing literature, showing that youth susceptibility to smoking, experimentation, and current use varies by type of protobacco media channel exposure. Susceptibility and experimentation are associated with exposure to static tobacco advertising, with experimentation being mediated by perceptions of peer tobacco use. Current tobacco use is also associated with static advertising through increasing perceptions of peer tobacco use. In addition, current tobacco use is associated with both exposure to static advertisements through perception of peer tobacco use of tobacco products, and by exposure to tobacco use in TV and movies, both directly and through perception of peer tobacco use.

Glantz and colleagues report that the number of incidents of tobacco use depicted in top-grossing films fell from 2005 through 2010, but rebounded steeply in 2011 and continued rising during 2012 [35–37]. This increased exposure to tobacco images in movies and on TV comes at a time of ongoing exposure to static tobacco advertisements in newspapers and magazines, retail stores, and the Internet [19,23]. The culminating effects of exposure across all protobacco media channels increase youth susceptibility, cigarette experimentation, and current tobacco use.

Both static advertising and TV and movie exposure to tobacco products operate indirectly by increasing perceptions of peer smoking, an important finding in this study. With few exceptions, analyses of the effects of exposure to onscreen smoking only consider direct effects of this exposure and treat peer influences as a separate independent variable [6,7, 38]. These findings suggest that estimates of population attributable risk for youth smoking associated with smoking depicted in movies could be underestimated if computed without considering the indirect effect of perceived peer tobacco use.

There are limitations to this study. First, the cross-sectional design of NYTS prevents causal inferences. However, findings are consistent with the existing literature demonstrating a dose-response relationship between exposure to depictions of smoking in movies and youth smoking [3]. Second, these data are self-reported. While less resource-intensive to collect, self-reported measures are subject to recall bias and reverse causality, and are less sensitive measures of exposure than other more resource-intensive techniques [39]. The consistency of our findings with studies that use more objective, resource-intensive measures of exposure demonstrate the utility of self-reported measures. Third, the NYTS captures exposure to tobacco use in TV and movies in one survey item, preventing exploration of the differential effects of each media type. Fourth, the survey item measuring exposure to tobacco advertising on the Internet does not specify source of advertising; thus, it may include recall of exposure to content or videos not promulgated by the tobacco industry. As Internet content becomes increasingly interactive, further examination of the specific types of online pro-tobacco influences viewed and research on how these Internet exposures may influence youth use is warranted.

In conclusion, this nationally representative study of US middle and high school students demonstrates that static tobacco advertising and exposure to onscreen tobacco images in TV and movies increase youth tobacco use in dose-response fashion. Estimates that fail to adequately account for indirect effects of static ad exposure and exposure to tobacco use depicted in TV and movies through perceived peer smoking may underestimate the total effects of protobacco media. Consistent with earlier research, this study finds that the effects of exposure to onscreen smoking depicted in TV and movies and the effects of exposure to static advertising are both critical to consider in youth tobacco use [9,40]. The results underscore the importance of efforts to reduce youth tobacco exposure in TV and movies, and through static advertising.
